# A Novel Quality Measure and Correction Procedure for the Annotation of Microbial Translation Initiation Sites

**DOI:** 10.1371/journal.pone.0133691

**Published:** 2015-07-23

**Authors:** Lex Overmars, Roland J. Siezen, Christof Francke

**Affiliations:** 1 Centre for Molecular and Biomolecular Informatics, Radboud University Medical Centre, Nijmegen, the Netherlands; 2 Netherlands Bioinformatics Centre (NBIC), Nijmegen, the Netherlands; 3 HAN BioCentre, University of Applied Sciences Arnhem Nijmegen, Nijmegen, the Netherlands; Academia Sinica, TAIWAN

## Abstract

The identification of translation initiation sites (TISs) constitutes an important aspect of sequence-based genome analysis. An erroneous TIS annotation can impair the identification of regulatory elements and N-terminal signal peptides, and also may flaw the determination of descent, for any particular gene. We have formulated a reference-free method to score the TIS annotation quality. The method is based on a comparison of the observed and expected distribution of all TISs in a particular genome given prior gene-calling. We have assessed the TIS annotations for all available NCBI RefSeq microbial genomes and found that approximately 87% is of appropriate quality, whereas 13% needs substantial improvement. We have analyzed a number of factors that could affect TIS annotation quality such as GC-content, taxonomy, the fraction of genes with a Shine-Dalgarno sequence and the year of publication. The analysis showed that only the first factor has a clear effect. We have then formulated a straightforward Principle Component Analysis-based TIS identification strategy to self-organize and score potential TISs. The strategy is independent of reference data and *a priori* calculations. A representative set of 277 genomes was subjected to the analysis and we found a clear increase in TIS annotation quality for the genomes with a low quality score. The PCA-based annotation was also compared with annotation with the current tool of reference, Prodigal. The comparison for the model genome of *Escherichia coli* K12 showed that both methods supplement each other and that prediction agreement can be used as an indicator of a correct TIS annotation. Importantly, the data suggest that the addition of a PCA-based strategy to a Prodigal prediction can be used to ‘flag’ TIS annotations for re-evaluation and in addition can be used to evaluate a given annotation in case a Prodigal annotation is lacking.

## Introduction

The *ab initio* identification of coding sequences is the first step in the annotation of a genome. Various computational methods have been developed to identify coding sequences from Open Reading Frames (ORFs) with low error rate. Automated identification of the Translation Initiation Sites (TISs) associated with the protein-encoding genes has proven to be more difficult. The difficulty probably relates to the fact that the sequence signatures that are associated with the initiation of translation can be diverse. In prokaryotes, the translation of the majority of protein-encoding genes is initiated by the interaction between a short sequence in the 5’ untranslated region (5’-UTR) of the mRNA, referred to as the Shine-Dalgarno (SD) sequence [1], and the 3’-end of the 16S ribosomal RNA. It was observed that the presence of the SD sequence is correlated with a higher expression level [2]. Similarly, the presence of the SD sequence correlated with the occurrence of an AUG codon as the translation start [2]. Nevertheless, the SD sequence is not absolutely required as it was found that many, and even some highly translated, mRNAs lack a (recognizable) SD sequence [3]. So far, two alternative (i.e., SD-independent) mechanisms of translation initiation have been identified [4]. The first SD-independent mechanism involves ribosomal protein S1 (RPS1), which interacts with the 5’-UTR to initiate translation [5]. The second mechanism involves the 70S ribosome as a whole, which can interact directly with leaderless genes (genes without a 5’ UTR) and uses an N-formyl-methionyl-transfer RNA to initiate translation [6,7]. The start codon is assumed to be the most important signal for the translation of leaderless genes. Analysis of 162 completed bacterial genomes showed that the number of genes not preceded by an SD-sequence is highly variable between bacteria, where the reported number varies between 9.2% and 88.4% [8,9].

Currently the most widely used gene-calling tools are GLIMMER3 [10] and Prodigal [11]. Other tools include MED2.0 [12], GeneMarkHmm [13] and EasyGene [14]. The former tools predict coding sequences with relative low error rates for genomes of well-studied organisms. Nevertheless, the annotation of genes in high-GC-content genomes using these tools is more challenging, since the genomes contain fewer random stop codons leading to longer Open Reading Frames (ORFs) and more mistakes [11]. Three main approaches are in use to improve upon a given TIS annotation. These are essentially based on: i) post-processing of initial predictions; ii) comparative genomics; and iii) combining multiple predictions. The related tools commonly start from existing genome annotations or genes identified by the before-mentioned prediction tools. For instance, TICO [15] was developed to improve the accuracy of TIS annotation by performing an unsupervised classification of strong-TIS and weak-TIS sequences. Similarly, various resources such as ProTISA [16] and SupTISA [17] have accumulated (post-processed) predictions from different sources. In ORFcor, orthologous sequences are used to identify and correct inconsistencies in the gene and TIS annotation [18]. Likewise, Genome Majority Voting was used to assign TISs based on groups of orthologous sequences [19]. The pipeline GenePRIMP [20] was developed to improve the gene prediction of bacterial genomes and to report anomalies including inconsistent start sites, and missed and split genes. Multiple gene-prediction methods have been combined to improve the accuracy of gene and TIS annotation [21–24]. It was found that the application of a specific path in the combination of predictors can provide a gain in sensitivity while maintaining a high specificity in gene prediction [21]. Nevertheless, a recent comparison of the various available prediction tools and pipelines indicated that the best performers achieved a maximal TIS prediction accuracy of around 90% for a typical genome [11]. Moreover, the addition or combination of tools did not often lead to an improvement in the estimated quality above 90%.

Different types of errors are commonly introduced by computational gene calling and annotation methods. First, true coding regions can be overlooked. However, the percentage of missed genes is estimated not to exceed 5–10% [25]. Second, some predicted genes do not represent a true coding sequence [11,21]. Third, the assignment of the correct start codon (i.e., the translation initiation site (TIS)) can be erroneous. Bakke and colleagues [26] evaluated the performance of three automated genome annotation services for the annotation of the archaeon *Halorhabdus utahensis*, namely: IMG [27], RAST [28] and the J. Craig Venter Institute (JCVI) Annotation Service [29]. There appeared to be considerably more agreement concerning the identified translation stop codons (90% shared) than concerning the annotated TISs (48% shared) between the three services. The inconsistency in TIS annotation was also highlighted by another study, in which it was shown that 53% of the orthologs among 5 *Burkholderia* genomes have inconsistently annotated TISs in RefSeq [30]. The incorrect annotation of TISs can flaw different types of genome analysis such as: the (automated) identification of regulatory sequences, the construction of reliable phylogenetic trees for homologous genes/proteins, the function annotation of the gene product and the prediction of the subcellular location of the gene product.

An important limitation in *de novo* gene prediction is the need for reference data-sets with correctly identified TISs to test the quality of annotations. Unfortunately, large sets of translated proteins where the N-terminus has been experimentally verified are scarce [31]. A frequently used dataset of verified protein sequences is available for *Escherichia coli* K12 MG1655 from EcoGene [32]. The translation start sites (926) in this dataset are reported to be experimentally determined using N-terminal protein sequencing. In this paper we present a strategy that avoids the need of reference datasets to assess the accuracy of genome-wide TIS annotation. The strategy involves a comparison between the distribution of alternative TISs around the annotated TISs within a genome, and an expected distribution that can be calculated based on simple and transparent criteria. Such a comparison appeared to provide an intrinsic quality metric for genome-wide TIS-prediction accuracy. We have evaluated the TIS quality for all sequenced genomes and found that the majority was reasonably well annotated, but a substantial minority (~13%) clearly needs to be improved.

In addition, we have developed an iterative Principle Component Analysis (PCA)-based strategy that uses the sequences surrounding all putative TIS for a gene, to identify the most likely TIS. The strategy neither involves training nor reference data, and is not based on any additional assumptions. It can thus be used for any genome. We have implemented the strategy and assigned TISs to all genes for a set of 277 representative bacterial genomes. Comparison of the TIS annotation for the *E*. *coli* K12 MG1655 genome as obtained with the PCA-based method to the annotation obtained using the standard tool Prodigal revealed a clear advantage of using both methods simultaneously.

## Results

### An inherent metric to assert the quality of genome-wide gene-predictions

**Fig 1:**
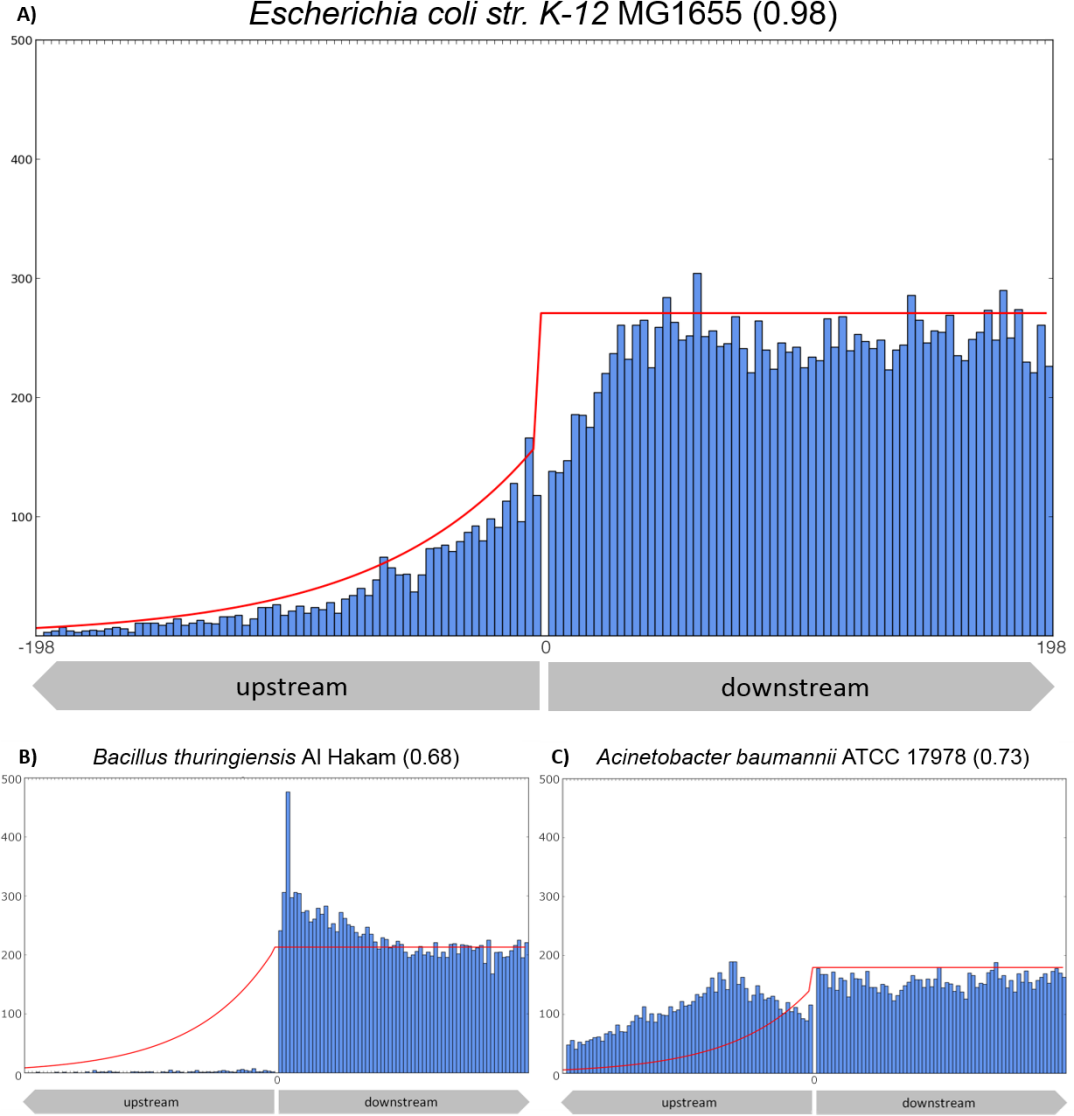
Three typical distributions of alternative start codons found for genomes in the NCBI RefSeq database. (A) The distribution of alternative starts in *Escherichia coli* K12 MG1655; (B) *Bacillus thuringiensis* str. Al Hakam; and (C) *Acinetobacter baumannii* ATCC 17978. For all ORFs that included an annotated gene and TIS, the total number of alternative start codons for each codon position relative to the annotated translation start were counted. The green line represents the expected distribution as determined using formula 1 In genomes that adhere to Fig 1A the observed and expected distribution are alike, whereas for genomes that adhere to B or C the observed distribution of alternative start codons given the annotation is clearly deviating from the expected distribution (green line). A comparison of the observed and expected distribution provides an inherent quality measure for genome-wide gene-prediction accuracy.

We identified all alternative in-frame translation initiation sites (TISs) for the annotated genes in the complete archaeal and bacterial genomes available via NCBI in January 2013 (see methods). We plotted the distribution of the position of all alternative TISs with respect to the annotated TISs (dataset available at Figshare; http://dx.doi.org/10.6084/m9.figshare.1460717). For the well-studied bacterium *E*. *coli* K12 MG1655 we found a characteristic distribution, where the number of alternative TISs in the coding part of the gene was reasonably constant and where the number decreased nearly exponentially upstream of the annotated start (Fig 1A). Furthermore we observed that in *E*. *coli* K12 MG1655 the first 5 to 10 codons of the coding sequence showed a relative underrepresentation of alternative TISs. In fact, the genomes of other well-studied bacteria such as *Bacillus subtilis* str. 168, *Lactobacillus plantarum* WCFS1, *Listeria monocytogenes* EGD-e, *Pseudomonas putida* KT2440, *Mycobacterium tuberculosis* M37Rv and *Salmonella typhimurium* LT2, showed a very similar distribution (S1 Fig). Moreover, the distribution of *Bacillus subtilis* str. 168 showed a characteristic peak of alternative starts 3 codons upstream from the annotated TIS. The same peak was observed for the other species of the phylum Firmicutes (S1 Fig panels D,C,F). We have also determined the distribution of alternative TISs in *Saccharomyces cerevisiae* S288C (S1 Fig panel H). The distribution of alternative TISs in this eukaryote appeared highly similar to the ones of the well-studied bacteria. At the same time, we found that a considerable number of genomes (~13%) showed a dissimilar distribution. The dissimilar distributions were of two types: i) a distribution that suggested alternative TISs upstream of the annotated TISs were absent (Fig 1B); and ii) a distribution that suggested that there was a relatively low probability to find a stop codon upstream of a TIS (Fig 1C). The former distributions commonly showed a peak of alternative TISs in the first 10 codons of presumed coding sequence. The latter distributions showed a peak of alternative TISs around 30 to 120 nucleotides upstream of the annotated TISs.

**Fig 2:**
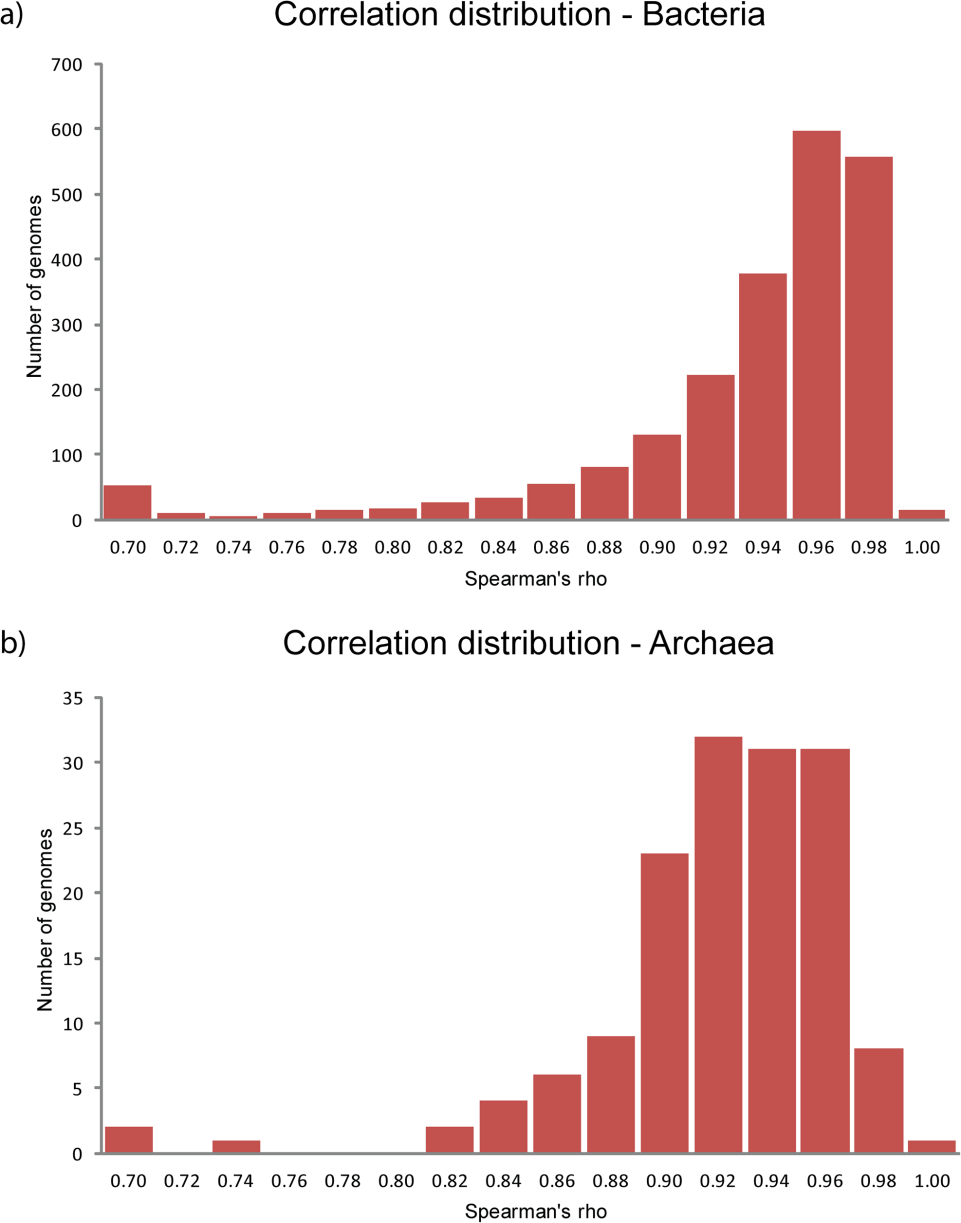
Correlation coefficients between observed alternative start frequencies and expected alternative start frequencies for microbial genomes. (A) Spearman’s rho coefficients for all bacterial RefSeq genomes with > 500 ORFs. (B) Spearman’s rho coefficients for all Archaeal RefSeq genomes with > 500 ORFs.

While studying the relative positions and frequencies of alternative in-frame TISs we realized that the overall distribution of the alternative start codons should in fact be a very good measure of TIS annotation quality. The distribution should ideally follow the distributions as found in the well-studied organisms, which all displayed a near exponential decrease upstream of the annotated TISs. We calculated the expected distribution for every individual genome given two simple premises: i) the probability of finding alternative TISs in coding sequence is constant on average and likewise in non-coding sequence; and ii) the probability of finding an in-frame stop-codon upstream of the TIS is constant on average. To take variation in AT or GC content in account we calculated genome specific in-frame alternative start codon frequencies and genome specific stop codon frequencies (see methods). The difference between the observed and the calculated distribution could then be used directly as a quality measure of TIS annotation. To probe the difference between the given and the expected distribution we decided to use a correlation measure since such a measure should be relatively insensitive to deviations at particular codon positions. We calculated a Spearman correlation coefficient between the given and expected distribution for all sequenced genomes in the NCBI RefSeq database of January 2013. The calculated correlations of both the upstream and the complete distribution for all analyzed genomes can be found in S1 Table. We observed striking differences between the given and expected distribution of alternative TISs for various genomes and found that these differences were more prominent in the upstream region. We therefore decided to use only the upstream correlation for comparison. Based on our two simple premises the calculated correlation coefficient should be a good measure of TIS annotation quality. Indeed the genomes known to be well annotated, like those of *Escherichia coli* K12 MG1655 and *Bacillus subtilis* str. 168, showed a high correlation coefficient (0.98 and 0.98, respectively). On the other hand, the distributions with a low correlation coefficient coincided with the a-typical distributions of alternative TISs similar to those depicted in Fig 1B and 1C. In case we used a correlation coefficient of >0.85 for genomes from 500–1500 ORFs and >0.9 for genomes from 1500 ORFs (see discussion) as indicative, the majority of genomes would be qualified as appropriately annotated (Fig 2). We found that in 88% of the bacterial genomes and in 71% of the archaeal genomes the TIS annotation quality measure was above the threshold (bacteria: 1936 of 2205, archaea 107 of 150).

### Factors that affect the quality of the annotation of TISs

**Fig 3:**
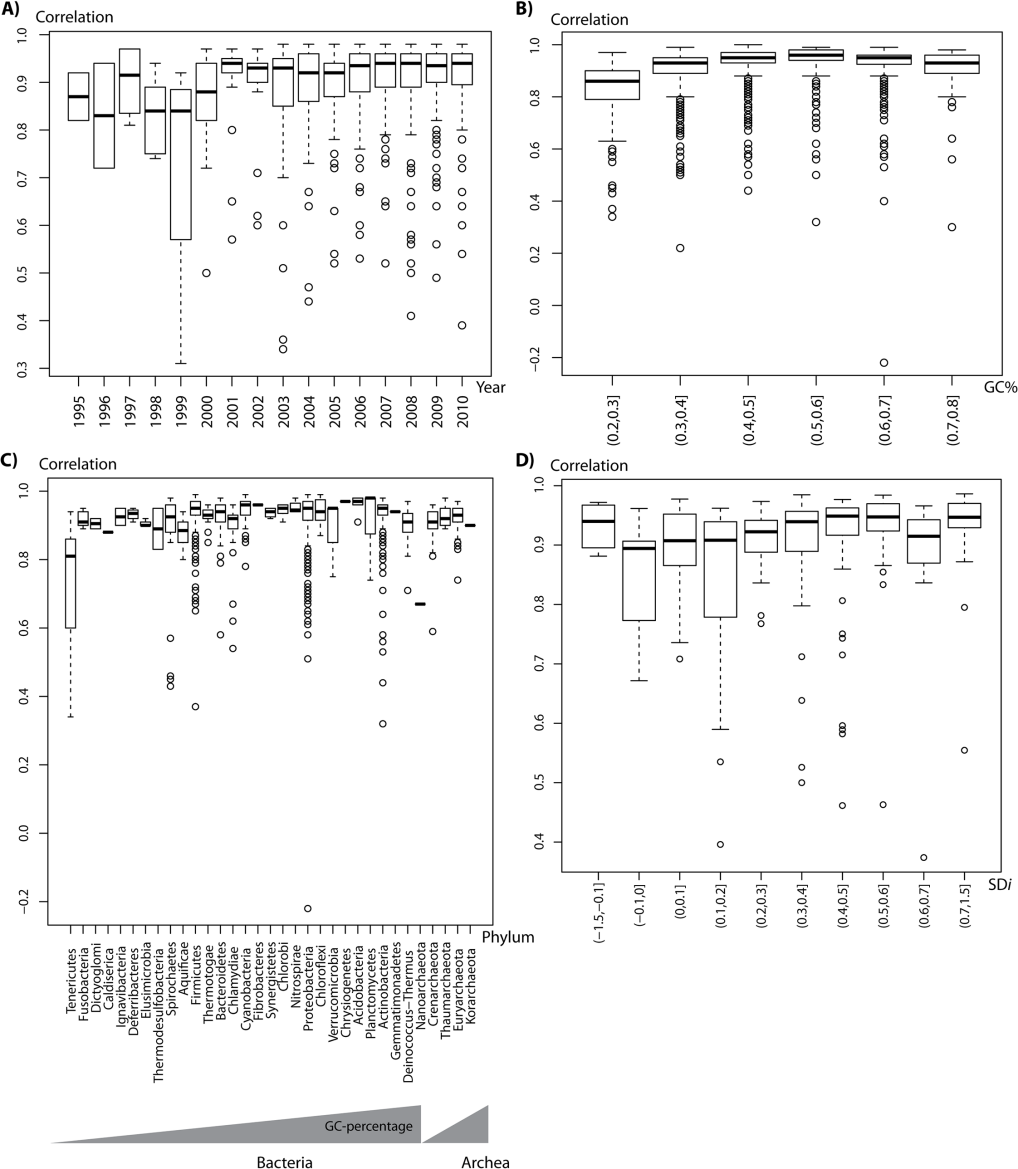
Effects of year of sequencing, GC-content and taxonomy on TIS-prediction accuracy. The boxplots show the distribution of the calculated correlation values (between the observed and expected distribution of alternative TISs) (*Y* axis) for: (A) all bacterial and archaeal RefSeq genomes grouped by year of sequencing (NCBI Bioproject data; [38]); (B) The RefSeq genomes grouped into 6 bins according to their GC%; (C) The RefSeq genomes grouped according to phylum; and (D) 277 selected bacterial and archaeal genomes with varying SD-index (proportion of Shine-Dalgarno sequence-preceded genes) [4].

The correlation between the given distribution of alternative TISs and the expected distribution was calculated per genome. An important consequence of this way of calculation was that it abolished the need for a reference gene-set and allowed a direct comparison of TIS annotation quality between genomes of varying GC content. For instance, we used the correlation measure to test the change in TIS annotation quality throughout the years. It has been assumed that the quality of the gene calling procedure, which includes the identification of TISs, has decreased in time due to the relative decrease in the number of manually curated annotations and the strong increase in the number of automated annotations [33]. Contrary to expectation, a comparison of the alternative TIS distribution correlation coefficients against the year of publication (Fig 3A) did not show such a trend.

Other factors, including GC-content, have also been proposed to be correlated to TIS annotation quality. We found that GC-content indeed correlated with the alternative TIS distribution correlation coefficients. Both high GC-content (>60%) and low GC-content (<40%) genomes showed a relatively low correlation between the given and expected alternative TIS distribution (Fig 3B). Using a Fisher exact test we determined that the occurrence of above-average quality gene annotation (correlation score > 0.85/0.9) for low and high GC-content genomes compared to the occurrence of high quality gene annotation in moderate GC-content genomes was significantly lower in all cases (p-value 0.0001 or smaller; S2 Table). We also observed a decreased correlation score for particular phyla (Fig 3C). However, the effect of phylum could be explained completely by the difference in the GC-content of the species within the phyla.

The number of genes preceded by a Shine-Dalgarno sequence within the genomes was another factor that we considered. We found that genomes with a lower SD presence on average had a lower TIS annotation quality (Fig 3D and S2 Fig). Yet, this tendency was not uniform as the group with the lowest SD presence (mostly Cyanobacteria and Bacteroidetes) had a relative higher TIS annotation quality. Vice versa, the group with a SD presence between 0.6 and 0.7 (mostly Firmicutes and Proteobacteria) had a relative decreased TIS annotation quality. Finally, we observed small differences in TIS annotation quality between genomes sequenced and annotated by the large sequencing centers (S3 Fig).

### The use of principal component analysis to identify TISs

Although the observed distributions of alternative TISs overall followed the expected distribution well, this was much less true for the sequence region directly upstream and downstream of the annotated TISs. For example, in *B*. *subtilis* str. 168 and *E*. *coli* K12 MG1655 a relative low number of alternative TISs were found in the first codons of the coding sequence, and peaks of alternative TISs were present in the upstream codons preceding the annotated TISs (Fig 1A and S1 Fig). In fact, the observed deviations should be expected in case recognition of the TIS requires a specific sequence signature. As a consequence, we reasoned, the true TISs should be separable from the alternative TISs based on the signature. Furthermore, the sequence signature related to translation initiation should stand out when the variability of the sequence directly upstream and downstream of potential TISs would be analyzed. The upstream and downstream sequences of all potential TISs for every annotated ORF in a particular genome were therefore converted to binary vectors (as described in the methods). A PCA was initiated using the vectors corresponding to the three longest potential gene-products for every ORF. Given the available data on model organisms (e.g., the *E*. *coli* reference set in EcoGene 3.0 [32]) the resulting set of vectors should represent a substantial number of true TISs (estimated number >20%) whilst ensuring that the majority of vectors represented false TISs (>66%). To enrich the set with true TIS corresponding vectors we iterated the PCA procedure (see methods). We found that the analysis converged within ten iterations for every genome analyzed. We thus have formulated an iterative PCA-based procedure to separate true TISs from alternative TISs (S4 Fig).

**Fig 4:**
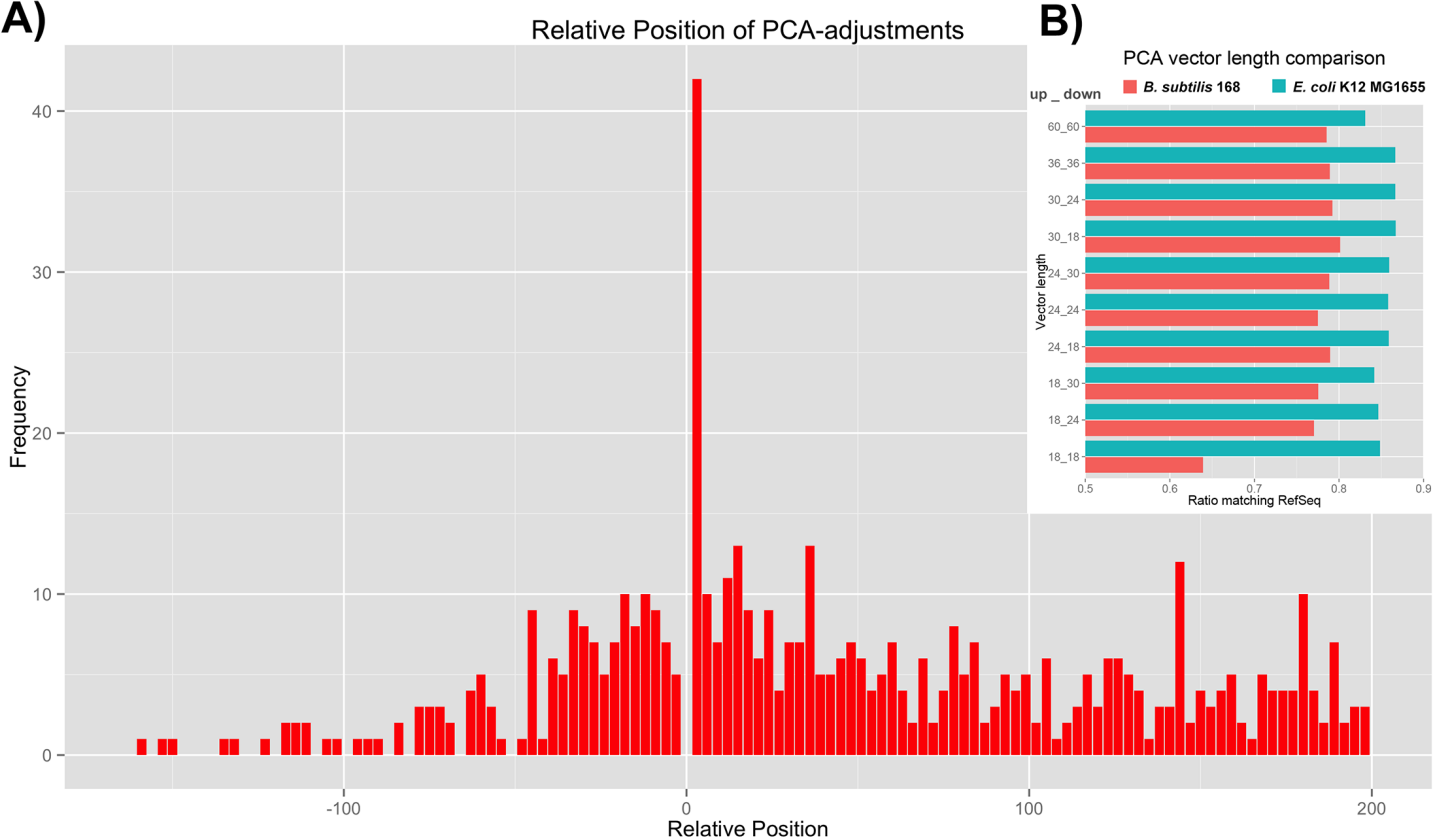
(A) The relative position of PCA-based TIS annotations that deviate from the RefSeq annotation for *E*. *coli* MG1655. (B) The effect of sequence vector length on the number of matching PCA-based and RefSeq TIS annotations in *E*. *coli* K12 MG1655 and *B*. *subtilis* 168. The following vector lengths were compared (denoted as: length upstream in nt. and length downstream in nt.): i) 60 & 60, ii) 36 & 36, iii) 30 & 24, iv) 30 & 18, v) 24 & 30, vi) 24 & 24, vii) 24 & 18, viii) 18 & 30 ix) 18 & 24 and x) 18 & 18.

We have applied the PCA-based procedure for *E*. *coli* K12 MG1655 and iteratively scored all potential TISs. We have included a table containing scores for the 5 best scoring TISs per gene for *E*. *coli* K12 MG1655 (S3 Table). The scripts that were used have been made available via Github (see methods) and can be used to evaluate the TIS annotation of any genome. When we employed the simplest assignment scheme, that is using the highest score achieved on principle component I during the iterations to discriminate the true TIS, ~85% of the TISs in *E*. *coli* K12 MG1655 were assigned identically when compared to the original annotation in the NCBI RefSeq database (Table 1). The majority of the non-compliant TIS annotations were located downstream of the annotated TIS, with a clear peak at the first downstream codon (Fig 4). To evaluate the optimal combination of upstream and downstream sequence lengths we performed the procedure for the phylogenetically distant model organisms *E*. *coli* MG1655 and *B*. *subtilis* str.168 using sequence vectors of varying lengths (see methods; the vector lengths are given in the caption to Fig 4). We found that for the reference genomes the best annotation results were obtained with sequence vectors that represented 30 nt upstream and 18 nt downstream of the annotated TIS (Fig 4B). We therefore decided to use sequence vectors of this length to annotate *E*. *coli* MG1655 (S4 Table), *B*. *subtilis* str.168 and a selected set of bacterial genomes

**Table 1:** TIS annotation for *E*. *coli* K12 MG1655. The NCBI RefSeq file contained 4141 annotated genes. The position of the TISs was compared between the PCA-based prediction, the Prodigal-based prediction and the RefSeq annotation. Recently, the EcoGene annotation has been updated and 13 TISs have been adjusted (b0259, b0552, b0656, b1994, b2030, b2192, b3218, b3505, b4543, b2803, b1331, b2982 and b3093). The adaptations were compared to the PCA-based and Prodigal-based predictions.

Annotation consistency^a^	Total	Verified set	Ecogene Adjusted	Ecogene adjustment
RefSeq = PCA = Prodigal	83% (3418)	88.4% (811)	1	12 nt upstream (b4543)
(RefSeq = Prodigal) ≠ PCA	9.8% (406)	7.8% (71)	0	
(Refseq = PCA) ≠ Prodigal	4% (173)	2.2% (20)	0	
RefSeq ≠ (PCA = Prodigal)	2% (88)	1.4% (13)	12	All in agreement with PCA = Prodigal
Refseq ≠ PCA ≠ Prodigal	1% (54)	0.2% (2)	0	

(a) The majority of TISs that are different in the PCA-based and Prodigal-based annotation are located close to the RefSeq TIS. For the PCA-based predictions: 548 were not in agreement with RefSeq, 199 of these where within 30 nt distance and 56 at 3nt distance; For the Prodigal predictions: 241 (6%) were not in agreement with RefSeq (and 74 (2%) were missed): 96 of these were within 30 nt distance and 30 at 3nt distance.

**Fig 5:**
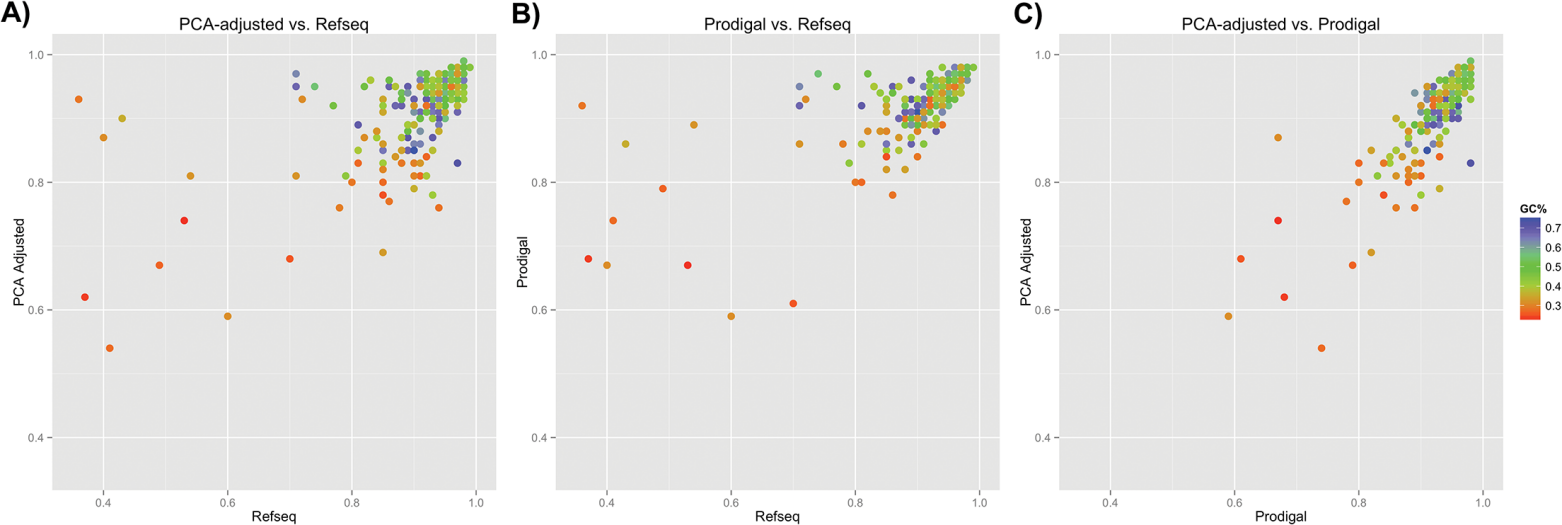
A comparison of TIS prediction accuracy between RefSeq, PCA-based and Prodigal annotation. Scatterplot of the correlation between observed alternative start codon frequencies and expected alternative start codon frequencies (i.e., the TIS annotation quality measure) for both the original TIS annotation as found in the RefSeq database (*Y* axis) and the adjusted annotations (*X* axis) based on (A) our iterative PCA pipeline and (B) Prodigal. (C) Scatterplot for PCA-based annotation versus Prodigal. The color scale represents the GC% of the corresponding genome (blue: high, green: average, red: low)

We applied the PCA-based annotation strategy to the 277 genomes selected by Nakagawa and colleagues [4]. The selection of genomes was made to provide a balanced representation of the bacterial and archaeal kingdom in terms of number of genomes per phylum. We found that using the iterative PCA procedure and simple scoring the calculated correlation of the distribution of alternative TISs with respect to the annotated start codon improved significantly for genomes with a poor correlation (and hence a poor TIS annotation quality) (Fig 5A). Only some of the high quality TIS annotations became slightly worse when applying our simple PCA-based ranking (see quality scores in S5 Table). Moreover, the quality of the PCA adjusted TIS annotation appeared to depend hardly on GC-content. An average quality measure of 0.91 was achieved, compared to an average score of 0.90 for the RefSeq annotations. The original genomic distributions of alternative TIS are supplied online (dataset available at Figshare; http://dx.doi.org/10.6084/m9.figshare.1460717).

For comparison, we also performed a *de novo* ORF annotation of the 277 bacterial- and archaeal- genomes using Prodigal [11]. Prodigal achieved a similar increase in the TIS annotation quality score with an average score of 0.92 (Fig 5B). Our PCA-based method and Prodigal showed a good correspondence in alternative TIS distribution correlation coefficients, where Prodigal performed only slightly better (Fig 5C and S5 Table). Moreover, we found that for almost all genomes Prodigal did not provide an ORF and TIS annotation for all ORFs of the NCBI annotation file. For various genomes the number of ORFs without matching Prodigal annotation exceeded 10% of the total.

To evaluate the differences in performance between the PCA-based TIS annotation and the TIS annotation by Prodigal in more detail, we compared the PCA-based annotation and the Prodigal annotation with the RefSeq annotation for the model organism *E*. *coli* K12 MG1655 and with the TIS annotation present in the well-curated Ecogene database (see Table 1). For the majority (82.6%) of genes the TIS annotation based on the PCA-based method and on Prodigal corresponded to the RefSeq annotation. For 406 (9.8%) genes the TIS annotation using the PCA-based method conflicted with the Prodigal and Refseq annotation. Vice versa, for 173 genes (4.2%) the PCA-based prediction was consistent with the RefSeq annotation but conflicted with that of Prodigal. These included 74 genes that were not called using Prodigal (e.g. no matching stop codon was found). Interestingly, for 88 genes (2.1%) the TIS annotation of both the PCA–based method and Prodigal were identical but conflicted with the RefSeq annotation. Moreover, we observed that for a large number of the genes in the latter group the distance between the annotated TISs was less than 30 nt (see Table 1 and Fig 4). Only in 54 cases (1%) all three annotations disagreed.

## Discussion

Two recent comparisons of the common gene identification algorithms showed that the algorithms mostly agree on the location of the genes but quite often provide an inconsistent positioning of the TISs [21,30]. Due to the availability of only a limited number of reliably curated genome annotations, TIS identifications might be biased. In fact, even for model organisms the number of datasets containing experimentally validated TISs is scarce [31]. The effect of bias on prediction quality is potentially underestimated given the fact that the identification algorithms have mostly been benchmarked using the same reference data.

We argue that quantification of the similarity between an observed (genome-wide) and the expected distribution of alternative TISs with respect to the annotated TISs provides an inherent measure of TIS annotation quality. The measure solely depends on the genome sequence that is being analyzed and is therefore reference independent. It is easy to establish, compare and interpret. We have implemented the proposed quality measure and found that in all the genomes assumed to have a high quality TIS annotation (i.e., reference genomes used in other papers) the observed distribution of alternative TISs corresponded well with the distribution that we calculated based directly on expected triplet frequencies. Therefore, the correlation between the observed distribution of alternative TISs and the expected distribution of alternative TISs appears indeed to be a good measure for TIS annotation quality. Moreover, the outcome of a similar analysis of TIS distribution for all chromosomes of the reference yeast *Saccharomyces cerevisiae* S288c (correlation: 0.98) suggests the quality measure can as easily be applied to eukaryotic genomes.

Our TIS distribution-based correlation measure was used to score TIS annotation quality for completely sequenced bacterial and archaeal genomes. Although a score of 1.0 reflects a perfect correlation, it must be noted that a somewhat smaller score probably already reflects the “perfect” score as the occurrence of some abnormal TIS sites could decrease the correlation slightly. Moreover, the number of alternative upstream TISs is in all cases relatively low–the number was lower than 150 directly upstream of the annotated TIS and decreased to zero within ~200 nucleotides for all genomes that were studied- and the related distribution should be relatively noisy as a consequence, thus reducing the correlation. Indeed, the well-annotated prokaryotic genomes of *E*. *coli* MG1655 and *B*. *subtilis* str.168 and the eukaryotic genome of *S*. *cerevisiae* S288c showed such a slightly reduced supposed optimal correlation of 0.98. We have used a correlation score >0.9 as indicative of appropriate TIS annotation. A correlation value >0.9 was obtained for all genomes with more than 1500 ORFs in the set of 277 selected genomes using either the original RefSeq TIS annotation, or the PCA-based annotation, or the Prodigal annotation (S5 Table). We observed that for smaller genomes the spread in correlation values became somewhat larger (S5 Fig). For genomes comprising 500 to 1500 ORFs therefore a correlation higher than 0.85 can be used as indicative of appropriate annotation.

We found that the genomes with a relative poor TIS annotation quality (500–1500 ORFs and score ≤0.85; >1500 ORFs and score ≤0.9) comprised about 13% of the genomes deposited in the Refseq database in January 2013. The TIS annotation of archaeal genomes is relatively more frequently of lower quality. For some genomes a very atypical distribution of alternative TIS was found, resulting in a very low annotation quality in the NCBI genome database. These genomes include for instance *Rhodospirillum photometricum* DSM 122 (0.22), *Rothia mucilaginosa* DY-18 (0.32), *Clostridium tetani* E88 (0.34) and *Borrelia turicatae* 91E135 (0.43). We have checked the corresponding publications to find abnormalities in gene-calling procedures and genome sequence characteristics and we found that indeed less common gene-calling procedures were used. Also genomes that are sometimes used as reference genomes were found to have a questionable annotation quality. For instance, the TIS distribution in *Streptococcus pneumoniae* R6 had a correlation of 0.83, whereas the correlation for other *Streptococcus pneumonia* genomes was >0.9. These observations underline the necessity to be careful in selecting a reference genome.

We have used the correlation measure to evaluate several factors that have been proposed to affect the TIS annotation quality. Contrary to expectation, we observed no correlation between the year the genome was published (annotated) and TIS annotation quality (Fig 3). This might well be related to the substantial increase in the quality of the annotation tools during the last decade. The sequencing center appears to have a small effect. Surprisingly, also the percentage of genes preceded by an SD-sequence in a genome does not seem to affect the TIS annotation quality much. In contrast, we found that genomes with low and high GC-content showed a significantly decreased TIS annotation quality. Thus low GC and high GC-content appear to be more problematic where the proper annotation of TIS by ‘traditional’ means is concerned.

Interestingly, the abundance of alternative TIS in the direct context of annotated TISs was aberrant. For example, in most, if not all, Firmicutes genomes we observed a characteristic peak of alternative TISs located 9 nucleotides upstream of the annotated TIS. The ribosomal binding site in these genomes explains this characteristic deviation. The full Shine-Dalgarno motif (AGGAGGU) needs only to be followed by a G to attain a GUG alternative start-codon. Genomes that belong to the Firmicutes phylum were among those reported to have the most genes preceded by a Shine-Dalgarno motif (up to 92%) [34]. In line with this, we observed that the majority of alternative starts in the observed peaks in the Firmicutes genomes are indeed GUG-codons. At the same time, relatively few alternative TISs were observed in the first codons of the coding sequence. Recent analysis of eukaryotic coding sequences also showed low numbers of AUG codons in the first 5–11 codons following the TIS. The low numbers were attributed to the prevention of translation of alternative genes [35]. Our observations for bacterial genes suggest that the low abundance of alternative start codons in the first part of the coding sequence is a universal trait of genes. Further work is under way to explore the possible biological relevance of the variability in codon abundance upstream and downstream of the TISs.

We have formulated a PCA based TIS scoring strategy and applied it to distinguish the true TISs from alternative TISs. An important advantage of the strategy is that it is self-organizing and that it thereby circumvents the need for reference data and knowledge of the characteristics of the genome that is analyzed. Thereby, every genome can be analyzed in the same way without impairing the overall quality. The PCA output can be used directly to manually assess the TIS annotation of individual ORFs.

A simple scoring scheme was applied to utilize the scores from the different iterations of PCA in an automated manner. We show that by applying the simple scoring scheme the TIS annotation quality of many of the relatively bad scoring genomes can be improved (Fig 5A). Moreover, The TIS annotation quality score observed after the PCA-based re-annotation of 277 representative bacterial genomes supports the reduced dependency between genome characteristics and TIS annotation quality, when compared to other predictors. We compared the quality of the PCA-adjusted TIS annotations with the ones derived from Prodigal and found that both methods improve the gene annotations in various genomes.

To evaluate the quality of the individual TIS annotations provided by the PCA-based strategy we compared the TIS annotation in *E*. *coli* K12 MG1655 provided by NCBI’s RefSeq database and by the Ecogene database [32], with the annotations calculated using our PCA-based strategy and Prodigal. We found that PCA-based- and Prodigal annotations were in agreement for the majority of genes (83%). The agreement is much better than the overall agreement in TIS annotation observed by [26] between different much-used annotation services, and the overlap observed for *Burkholderia* orthologs [30]. Importantly, the algorithms and information employed in our PCA-based strategy may be viewed as predominantly independent from the algorithms and information used by Prodigal. Whereas, Prodigal and most annotation procedures rely on the use of Hidden Markov Models and Dynamic Programming algorithms to score the TISs [36] and use reference data and the whole genome sequence to make sequence models, our procedure is devoid of such models and solely employs the sequence surrounding potential TISs and self-organizes those sequences using PCA. Therefore, the PCA-based strategy adds valuable independently obtained information to the Prodigal annotation.

In *E*. *coli* K12 MG1655, we found that in 2% of the cases the PCA-based and Prodigal predictions complied but were different from the Refseq annotation. Although the Refseq TIS annotation of *E*. *coli* is used as a reference, it does contain mistakes. For instance, in a recent update of the Ecogene database (Ecogene 3.0 [32]) 13 adjustments (with respect to the Refseq annotation) were made in the annotation. In fact, all of these were made to genes for which the PCA-based and Prodigal TIS predictions agreed, further suggesting that a compliance between the PCA-based and Prodigal annotation is a strong indicator for a correct TIS annotation. The fact that for the experimentally verified set of genes the correct prediction rate is even higher in the case of compliancy further supports the assertion. Moreover, recently the *fes* gene (b0585) was removed from the experimentally verified set [32] because the N-terminus of the encoded protein that was reported in literature before [37] was found 26 amino acids too short in a shotgun MS experiment [38]. The related TIS annotation, which was located 78 nt upstream of the TIS annotated in the Refseq database, was assigned correctly by both our PCA-based method and Prodigal. The incorrect annotation was corrected in the latest update of the *E*. *coli* K12 MG1655 Refseq record.

The above implies that consistency between a PCA-based annotation and Prodigal is a good indicator of proper TIS annotation and suggests that it will be useful to manually evaluate the TIS annotation in case the Prodigal/PCA-based annotation disagrees with the existing annotation. A difference between the PCA-based and Prodigal prediction would be another reason to ‘flag’ the annotation for manual curation. We found that for 14% of the *E*. *coli genes* either the PCA-based (406; 10%) or the Prodigal (173; 4%) TIS annotation were not in agreement with the RefSeq TIS annotation. Considering the numbers, adopting the Prodigal annotation instead of the PCA-based annotation would lead to a better annotation in the case of *E*. *coli*. However, the performance of both prediction strategies depends clearly on the genome that is being annotated as is implied by the data in Fig 5. Therefore a preference for one method over the other cannot be generalized. In addition, we observed that for a large number of the genes with a different TIS annotation between the PCA-based method and Prodigal the distance between the annotated TISs was less than 30 nt and was actually peaking at 3 nt (a single codon difference). Given the self-organizing nature of the PCA-based method it could be that these TIS in fact have been incorrectly annotated in RefSeq. Importantly, the Refseq and Ecogene database also contained a number of annotated genes that where not found using Prodigal. For 74% of these genes (55 out of 74) the PCA-based TIS annotation was the same as the one found in the reference database. This implies that the PCA-based TIS scoring strategy can be used to evaluate the TIS annotation for ORFs that are not recognized by Prodigal or other conventional gene prediction methods. In fact, we found that for various genomes Prodigal missed more than 10% of the ORFs reported in the RefSeq database (S5 Table).

The percentage of genes for which the PCA-based prediction was different from the Prodigal prediction and both different from the RefSeq annotation was around 1%. This small number implies that a combination of the sequence information used in the PCA-based method and the algorithms used in Prodigal together must capture most of the properties of the transcript sequence that determine the location of translation initiation sites in *E*. *coli*. In fact, the deviant TISs might be interesting to investigate in more detail because of their atypical character and thus because of a potentially alternative translation initiation mechanism.

### Conclusion

The newly defined distribution-based score for TIS annotation provides a powerful tool for the assessment of TIS annotation quality because it can be employed on any genome sequence without the need for a reference. We have evaluated the TIS annotation quality of the complete bacterial genomes present in the NCBI RefSeq database and found that a significant portion of genomes (~13%) has a questionable TIS annotation. To improve the quality of the genome annotation data in the public domain we therefore would consider it valuable that the TIS annotation quality is assessed before researchers publish their genome annotation. Fortunately, our analysis shows that despite the increased automation the overall TIS annotation quality has increased over the years.

We have developed an iterative PCA-based strategy to evaluate existing TIS annotations. The strength of the strategy is that it employs self-organization and is thus independent of reference data or *a priori* calculations. We have compared between PCA-based and Prodigal TIS annotations for the reference genome of *E*. *coli*. The analysis showed that both methods supplement each other and that an agreement between the methods is a strong indicator of a correct TIS annotation. Importantly, the addition of the PCA-based strategy to score potential TISs can also be used to ‘flag’ particular annotations for manual curation. Currently, the iterative PCA-based procedure only uses the positions on PCA component I to score TISs. Integrating scores based on specified features such as RBS sequence, coding/non-coding biases could potentially further improve the accuracy.

## Materials and Methods

### Genome sequences, annotations and sequencing meta-data

Genome sequence and annotation information of all bacterial and archaeal genomes was obtained from the FTP server of NCBI RefSeq (ftp://ftp.ncbi.nih.gov/genomes/Bacteria/) [39]. Genomes with less than 500 ORFs were excluded. The NCBI BioProject database was used to retrieve metadata on the sequencing projects, such as year of sequencing [40]. For all species with a sequenced genome that was published before October 2009, additional metadata such as sequencing center, were derived from the GOLD database [41]. The SD index, that is the fraction of genes preceded by a Shine-Dalgarno sequence, for a selected set of 277 bacterial and archaeal genomes was taken from Nakagawa et al. [4]. Taxonomic classifications were retrieved from the NCBI taxonomy database (ftp://ftp.ncbi.nih.gov/pub/taxonomy).

### Scripting and data analysis

All automatic procedures were written and executed in Python, whereas the Principal Component Analysis (PCA) and additional statistical analyses were written and executed in R3.0.0 [42]). The distribution plots of alternative TISs were generated using Matplotlib [43].

### Identification of alternative TISs in the context of prior annotation

The genomic position of the annotated translation initiation sites (TISs) for all genes within the microbial RefSeq genomes with >500 annotated open reading frames (ORFs) were taken from the.PTT files available at NCBI. The corresponding coding DNA sequences and upstream regions were collected. Alternative TISs were identified using the nucleotide triplets "AUG", "GUG" and "UUG" as potential start sites and the nucleotide triplets "UAA", "UAG" and "UGA" as stop codons. To identify potential alternative TISs the following criteria were applied: (i) the alternative TIS was in-frame with the annotated TIS; (ii) there was no in-frame stop codon located between the candidate TIS and the annotated TIS; and (iii) the alternative TIS was either found upstream or maximally 198 nucleotides downstream of the annotated TIS. For every genome the distribution of the genomic positions of the alternative TISs with respect to the annotated TISs was calculated. Simultaneously, an expectation of the distribution was calculated based on the sequence properties of the particular genome at hand. The number of alternative TISs in a window of 198 nucleotides upstream of the 3’ end of all annotated ORFs was used to calculate an expected frequency for the occurrence of alternative start codons in the coding part of any gene in the particular genome. A window of 198 nucleotides was chosen because for all studied genomes at this distance the expected total number of alternative in frame TISs has decreased below a total of 10. The expected start codon frequency of occurrence upstream of an annotated TIS (denoted as *f* ^*start*^(*obs_upstream*) was considered constant and was determined on basis of the average number of observed in-frame alternative starts (independent of in-frame stops) in a window of 198 nucleotides upstream of the longest possible ORFs (independent of the annotated TIS). The expected stop codon frequency of occurrence upstream of an annotated TIS was taken as 3 codons (UAA, UGA and UAG) in 64 and corrected for the AT and GC content of a genome (see *formula 1*). Using the frequencies derived above, we calculated the expected number of alternative TISs (n^*TIS*^) upstream at codon position *i* with respect to the annotated TIS, as described in *formula 2* (where N is total number of annotated ORFs):

$$f^{stop}(calc\_upstream)=([\text{fraction}(\text{GC})+\text{fraction}(\text{AT})/2]*\text{fraction}{\left(\text{AT}\right)}^{2})/4$$ formula 1

$$n^{TIS}(i)=N*f^{start}(obs\_upstream)*(1-f^{stop}(calc\_upstream))$$ formula 2

The similarity between the distribution of alternative starts-derived using the provided annotation- and the expected distribution of alternative starts-calculated on the basis of the genome sequence- was quantified using a Spearman’s rank correlation coefficient.

### Principal component analysis procedure to assess TISs

An iterative procedure of ten subsequent rounds of PCA was implemented to distinguish TISs on basis of common sequence patterns (procedure depicted in S4 Fig). For each identified candidate TIS in a genome a fixed number of nucleotides upstream and downstream of the annotated start codon were extracted from the sequence file. The upstream and downstream nucleotide sequences were fused to the first nucleotide of the corresponding start codon. The resulting nucleotide sequence was converted to a binary vector in which each position within the sequence was represented by four binary values corresponding to the four different bases (e.g., CTT was thus expressed as 0010 0100 0100). For each annotated ORF the TISs that resulted in the three longest ORFs were used as input for the initial round of PCA. We assumed such a selection included a sufficient number of true TISs to direct the initial PCA. After each round, all alternative and annotated TISs were projected on the linear combination of the first principle component (PC1) and the PC1-score for each candidate start was computed. The three top scoring candidate TISs for each ORF were then included in the next round of PCA. The number of iterations was set to 10, which was sufficient to converge the PCA results for all genomes tested.

## Supporting Information

S1 Fig Distribution of alternative start codons for well-studied bacteria and *Saccharomyces cerevisiae*. For all ORFs per genome that included an annotated gene and TIS, the total number of alternative start codons for each codon position relative to the annotated translation start were counted. The distribution of alternative start codons with respect to the annotated start in the RefSeq database are given for (A) *Bacillus subtilis* str. 168, (B) *Escherichia coli* K12 MG1655, (C) *Lactobacillus plantarum* WCFS1, (D) *Listeria monocytogenes* EGD-e, (E) *Mycobacterium tuberculosis* H37Rv, (F) *Pseudonoma Pseudomonas putida* KT2440, (G) *Salmonella typhimurium* LT2 and (H) *Saccharomyces cerevisiae* S288c.
(PDF)

S2 Fig Effect of Shine-Dalgarno presence on TIS-prediction accuracy. Scatterplot showing the relationship between TIS annotation quality (i.e. the correlation between observed alternative start codon frequencies and expected alternative start codon frequencies) (*Y* axis) and adjusted SD-index (proportion of Shine-Dalgarno sequence-preceded genes [4]) (*X* axis) for 277 bacterial and archaeal genomes with varying SD-index ([4]).
(TIF)

S3 Fig Effect of sequencing center on TIS-prediction accuracy. Boxplot of the distribution of TIS prediction accuracy values for the genomes in the GOLD database [39], grouped according to sequencing center.
(TIF)

S4 Fig Schematic overview of the iterative principal component analysis procedure to assess TISs. (TIF)

S5 Fig Relation between number of ORFs and annotation score. Scatterplot showing the relationship between TIS annotation quality (i.e. the correlation between observed alternative start codon frequencies and expected alternative start codon frequencies) (*Y* axis) and number of ORFs in a genome (*X* axis) for 277 bacterial and archaeal genomes ([4]).
(TIF)

S1 Table TIS annotation quality for all RefSeq genomes. The file contains the spearman rank correlation between observed alternative start codon frequencies and expected alternative start codon frequencies for each microbial RefSeq genome. In addition taxonomic information and the number of ORFs are given.
(XLSX)

S2 Table Fisher exact p-values for GC-content significance. The file contains the fisher exact p-values of the comparison of the TIS annotation quality (high: > = 0.9 or low: < 0.9) of moderate (bins: 0.4 < 0.5 & 0.5 < 0.6) GC% genomes to low- (bins: < 0.3 & 0.3 < 0.4) and high- (bins: 0.6 < 0.7 & > 0.7) GC% genomes.
(XLSX)

S3 Table Results of PCA procedure for *Escherichia coli* K12 MG1655. The file contains the results of the PCA-based scoring of TISs. Per ORF, a maximum of 5 potential TISs are included, for which the genomics positions, the positions on PC1, PC2 and PC3 and a rank based on the PC1 position are given for 10 PCA iterations.
(XLS)

S4 Table PCA-adjusted annotation for *Escherichia coli* K12 MG1655. The file contains the genomic positions and the PC1, PC2 and PC3 position in the 10 PCA iterations for each TIS that was selected using our PCA-based procedure. It also provides the TIS position predicted by Prodigal, the TIS position provided by RefSeq and the comparison among the 3 annotations (1 = True and 0 = False).
(XLSX)

S5 Table TIS annotation quality of RefSeq-, PCA-adjusted and Prodigal annotations for 277 species. The file contains the calculated quality measure of the TIS annotation based on the PCA-based prediction, the Prodigal-based prediction and the RefSeq annotation for the 277 species selected by Nakagawa and colleagues [4]. In addition, it provides the number of ORFs for which predictions were made and the GC% of each genome.
(XLSX)
