# Supplementary figures and images for: A Novel Quality Measure and Correction Procedure for the Annotation of Microbial Translation Initiation Sites
Source: PLoS One. 2015 Jul 23;10(7):e0133691. doi: 10.1371/journal.pone.0133691 (PMC4512697; doi:10.1371/journal.pone.0133691)

A)

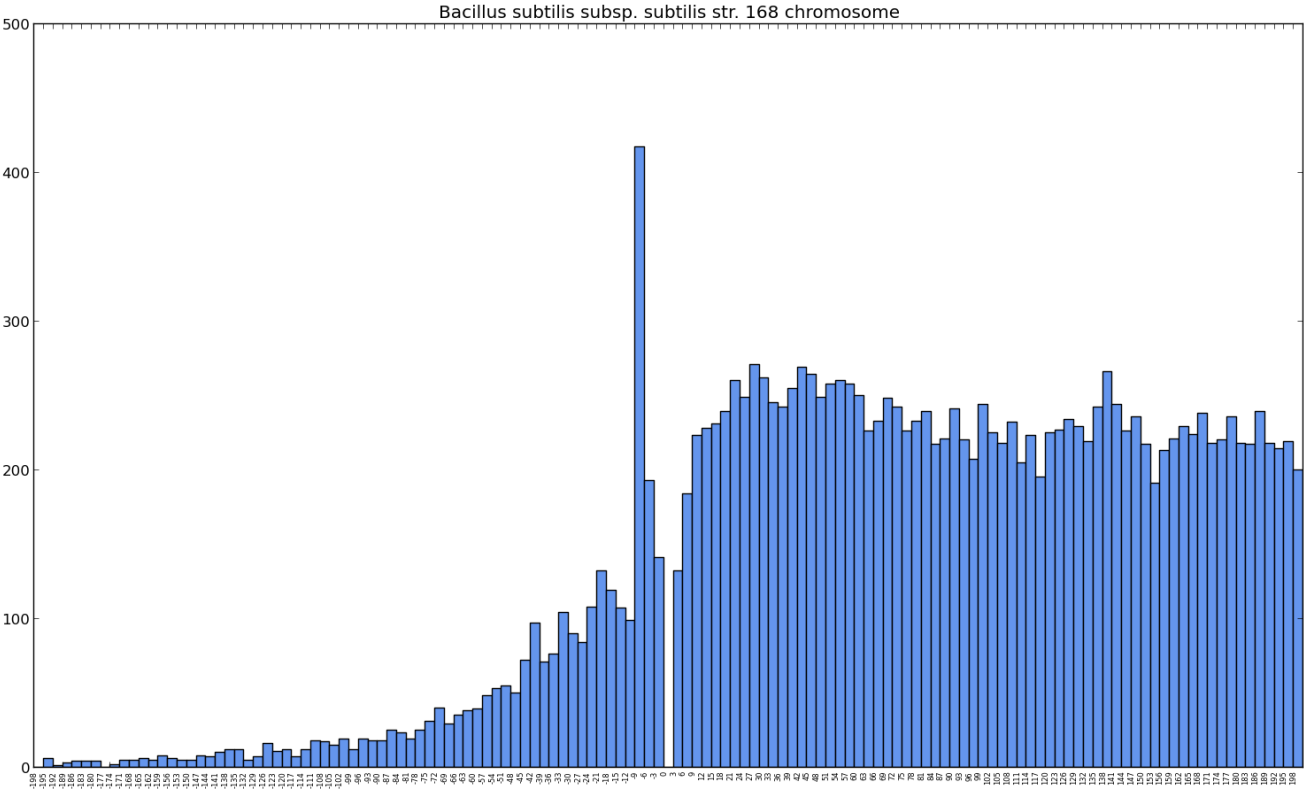

B)

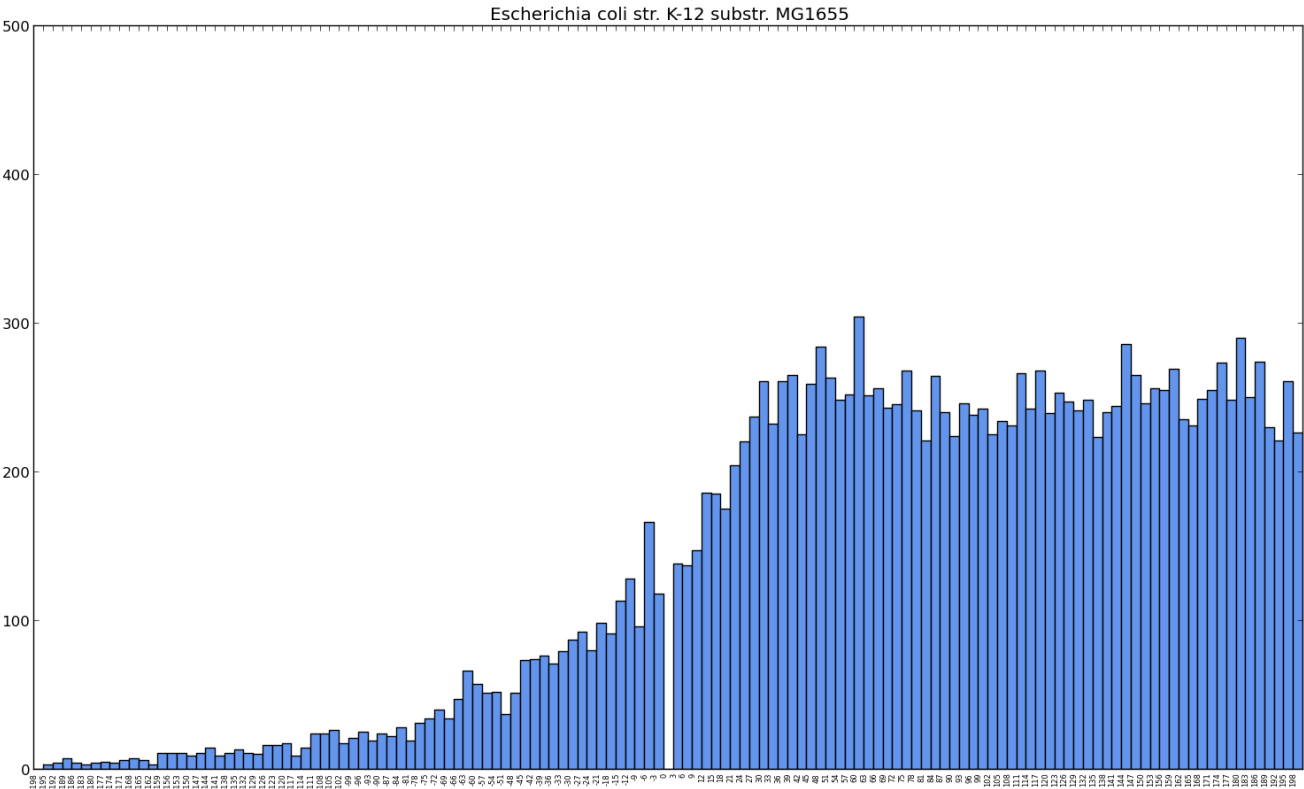

C)

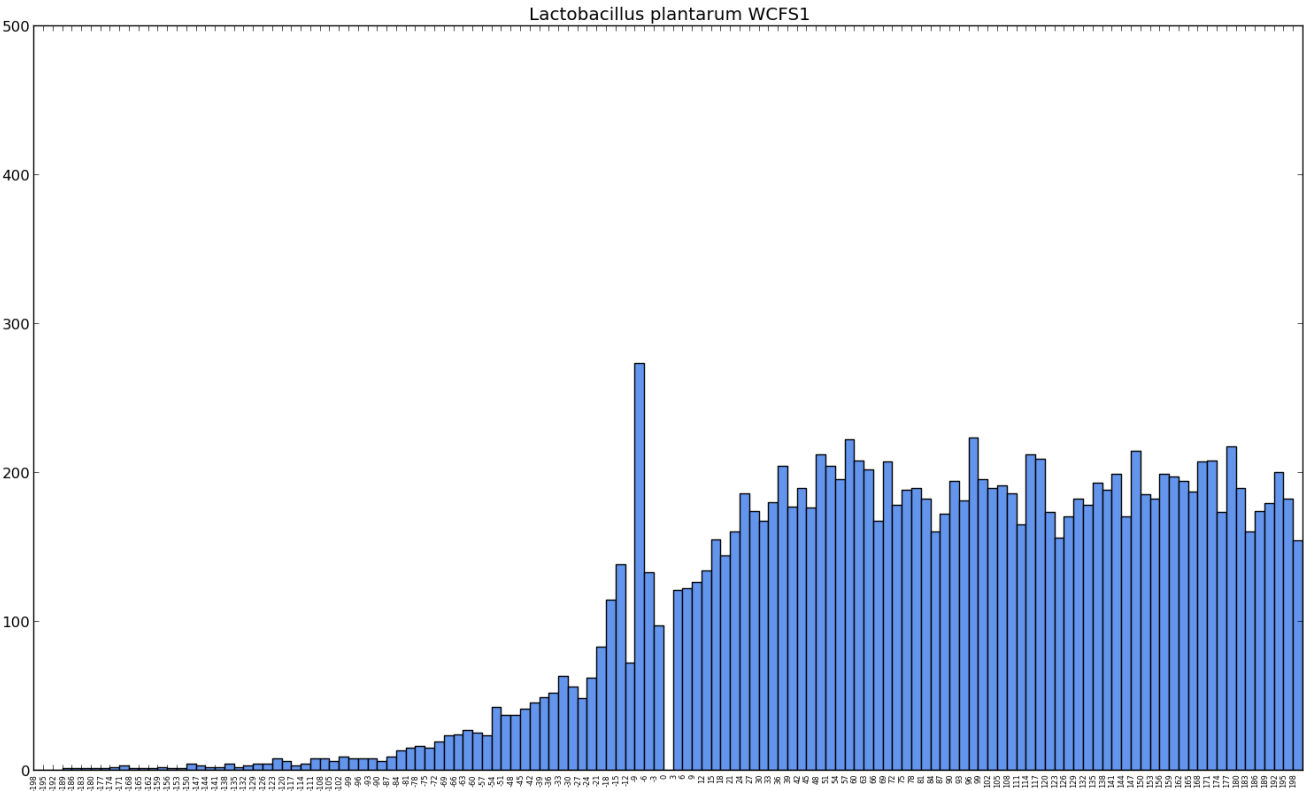

D)

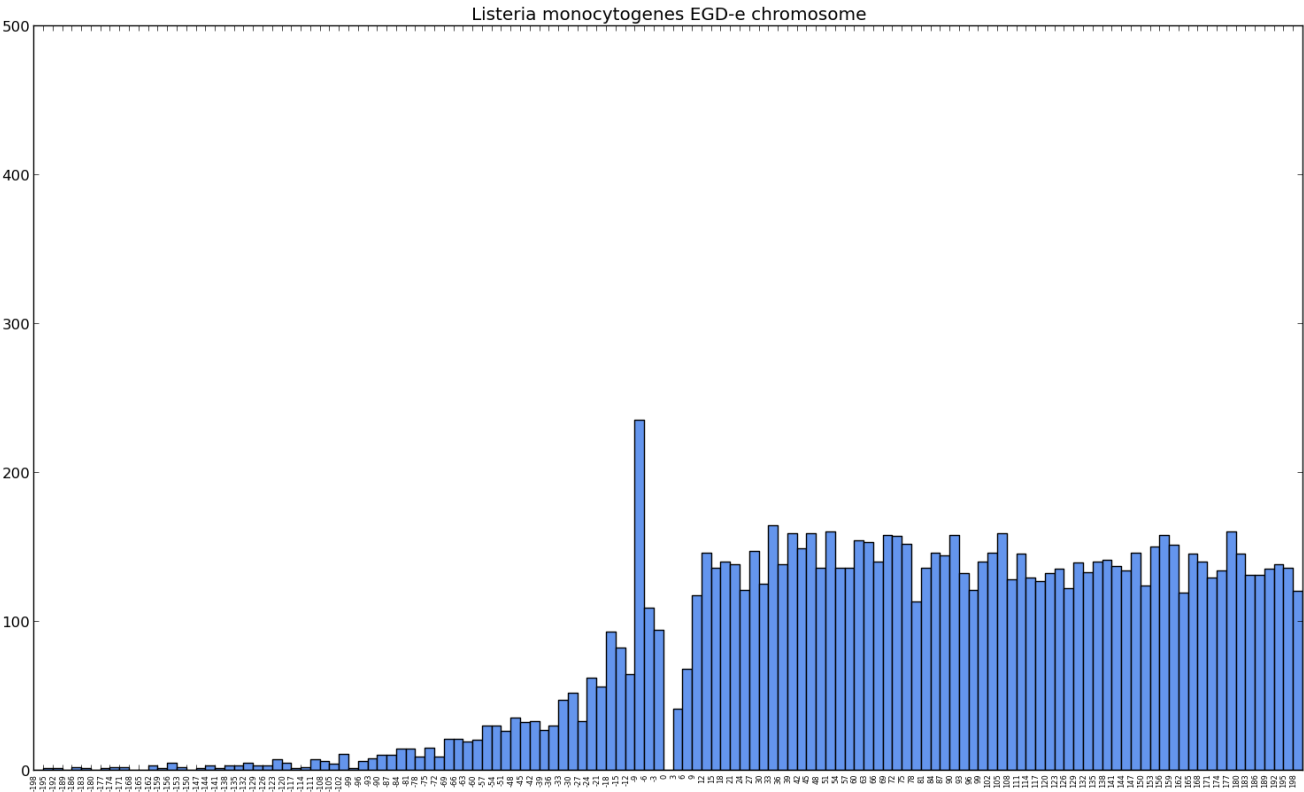

E)

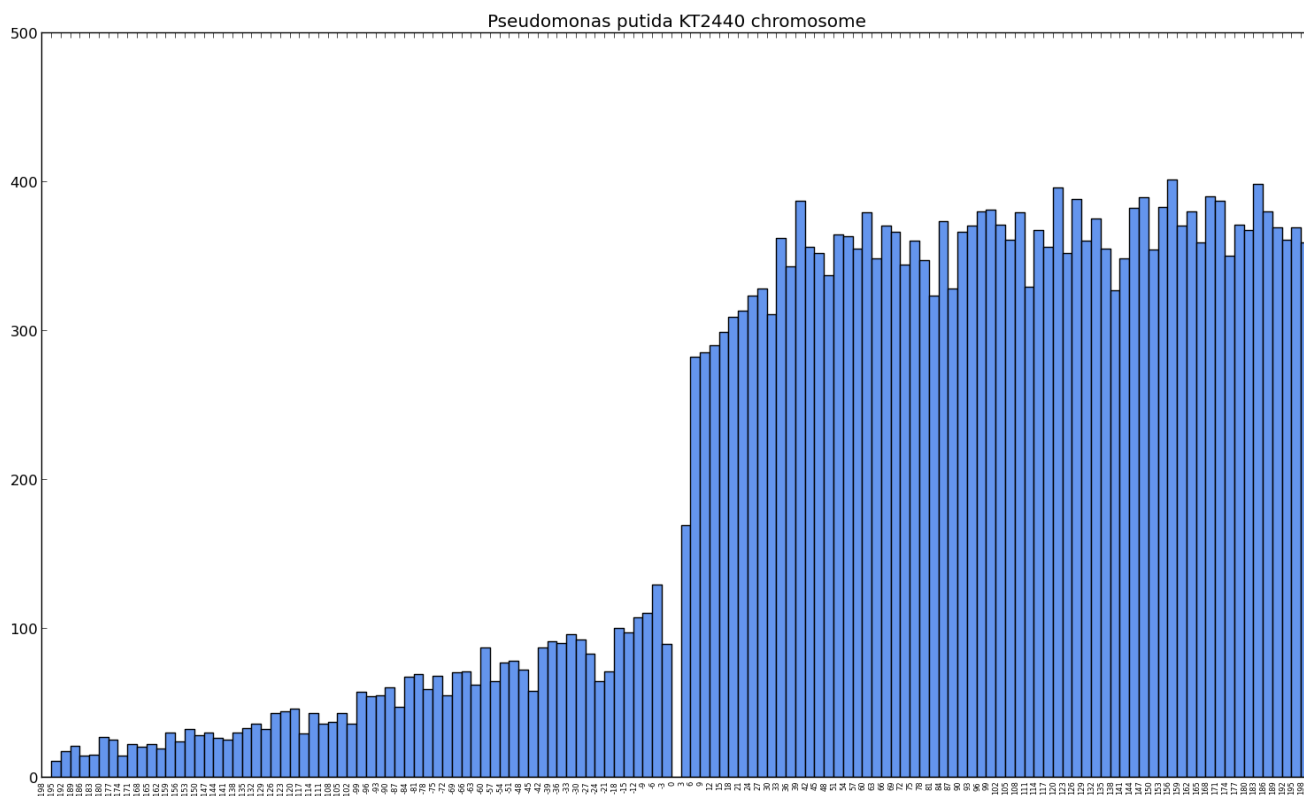

F)

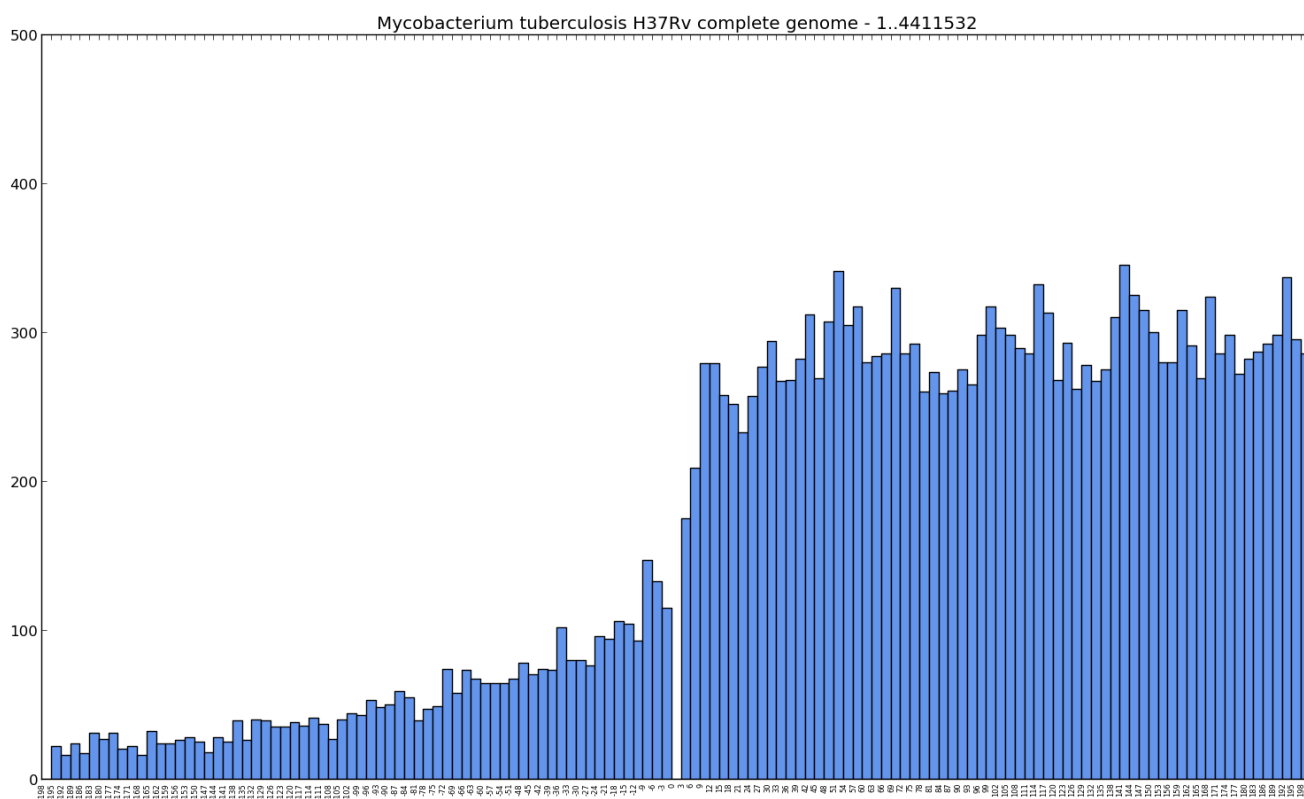

G)

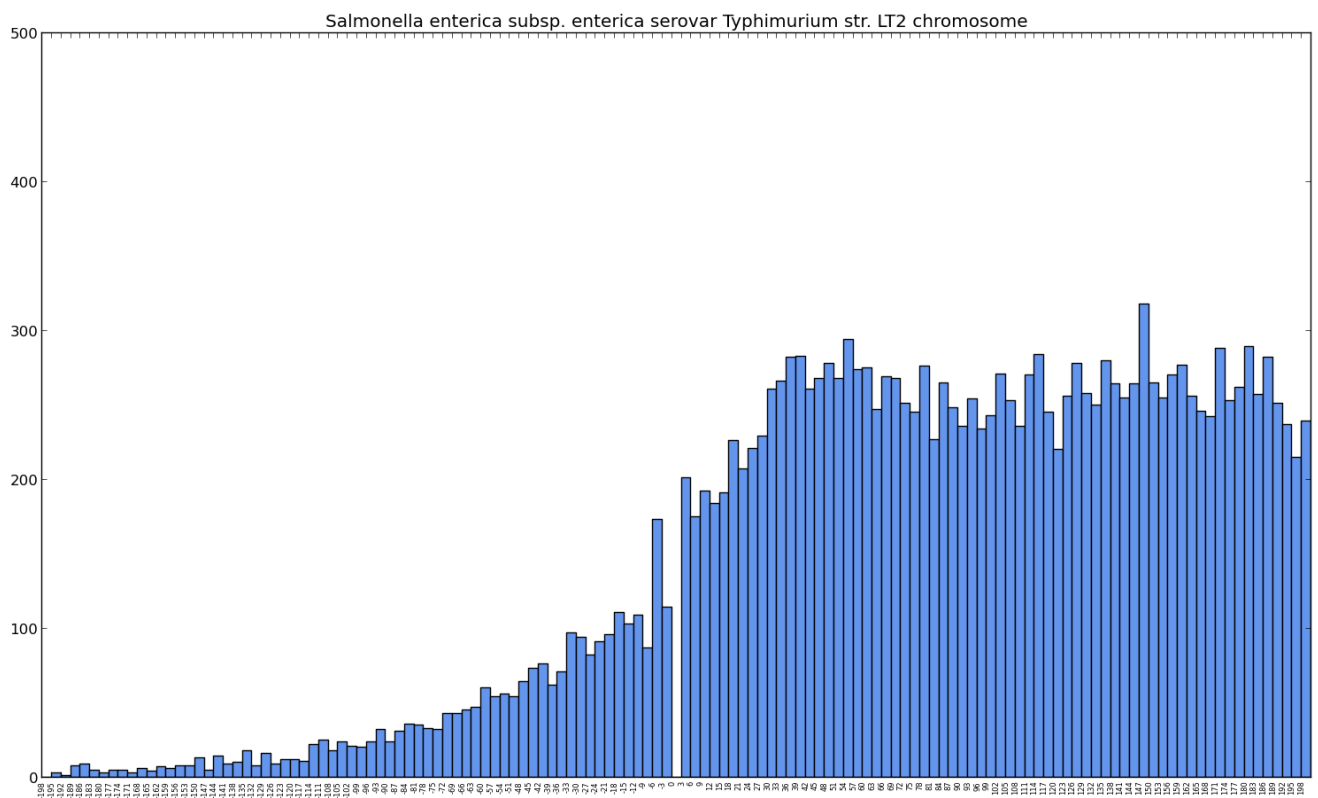

H)

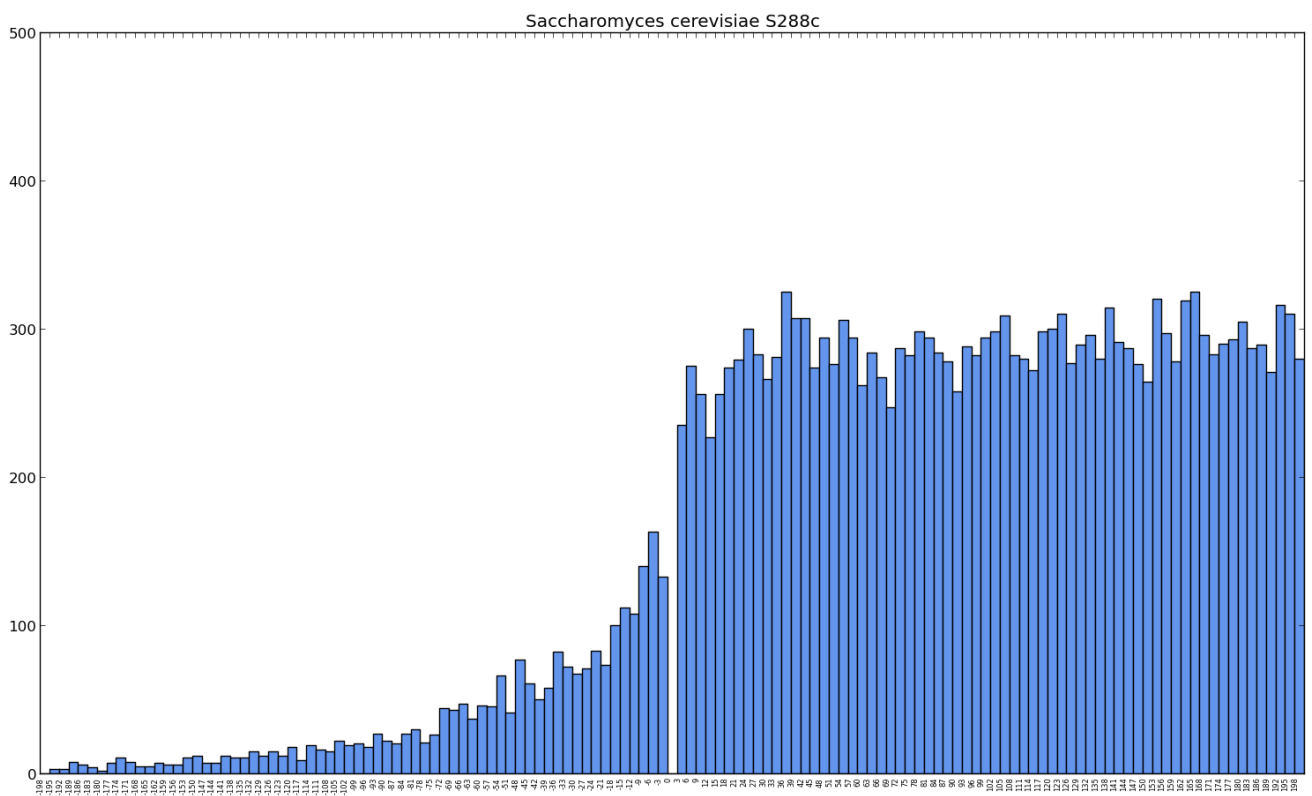

Supplement: S1 Fig — For all ORFs per genome that included an annotated gene and TIS, the total number of alternative start codons for each codon position relative to the annotated translation start were counted. The distribution of alternative start codons with respect to the annotated start in the RefSeq database are given for (A) Bacillus subtilis str. 168, (B) Escherichia coli K12 MG1655, (C) Lactobacillus plantarum WCFS1, (D) Listeria monocytogenes EGD-e, (E) Mycobacterium tuberculosis H37Rv, (F) Pseudonoma Pseudomonas putida KT2440, (G) Salmonella typhimurium LT2 and (H) Saccharomyces cerevisiae S288c. (PDF) [file pone.0133691.s001.pdf]

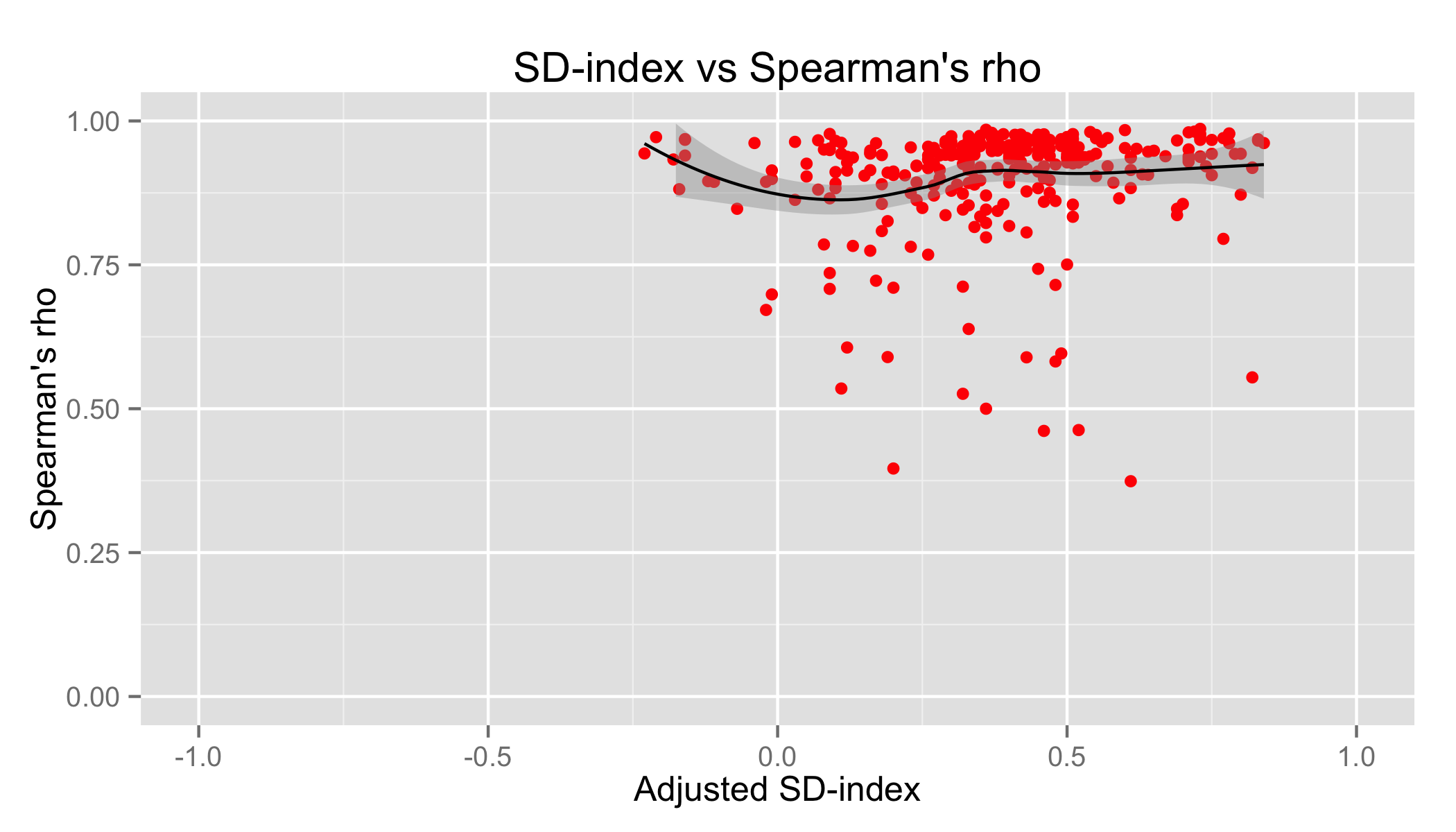

Supplement: S2 Fig — Scatterplot showing the relationship between TIS annotation quality (i.e. the correlation between observed alternative start codon frequencies and expected alternative start codon frequencies) (Y axis) and adjusted SD-index (proportion of Shine-Dalgarno sequence-preceded genes [4]) (X axis) for 277 bacterial and archaeal genomes with varying SD-index ([4]). (TIF) [file pone.0133691.s002.tif]

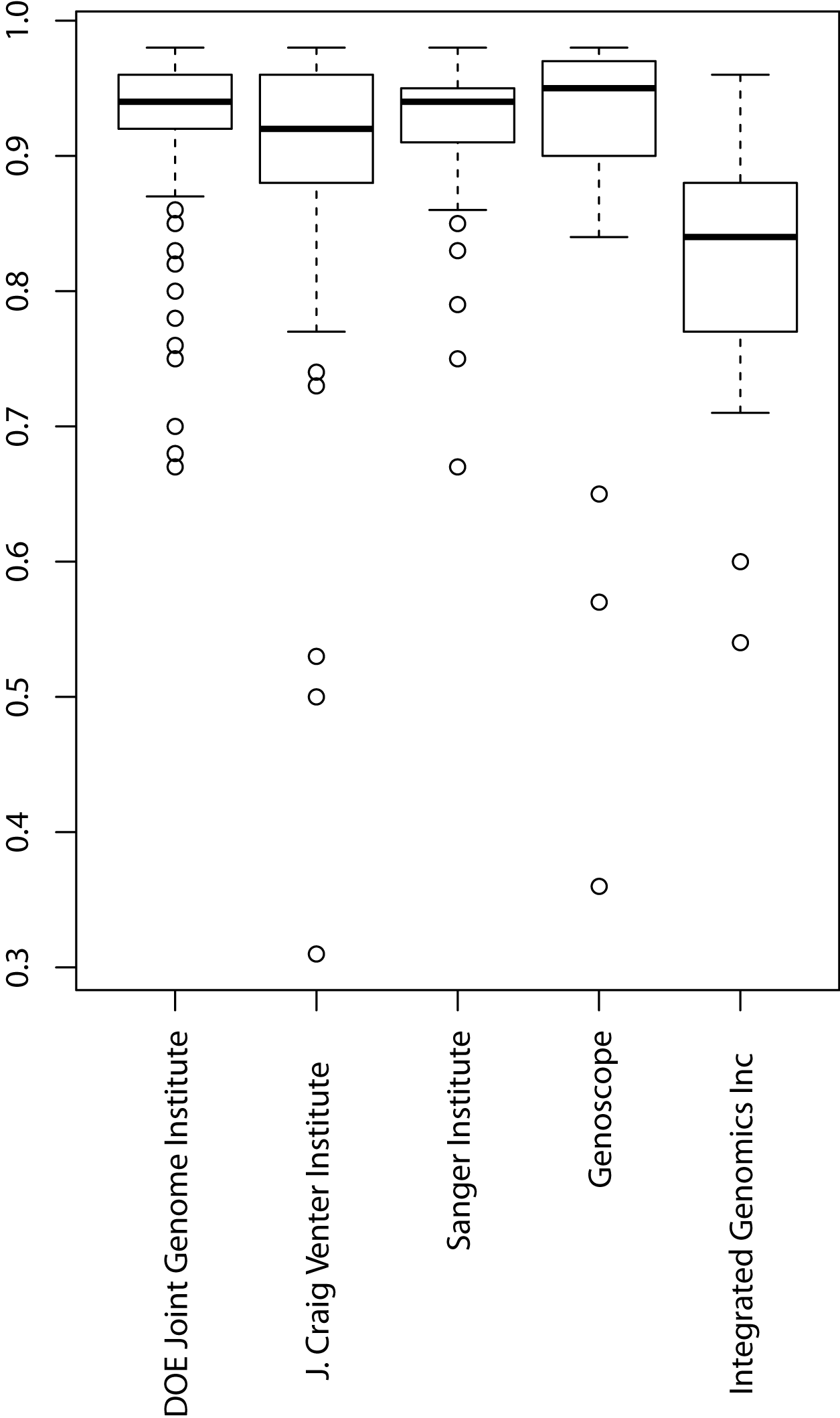

Supplement: S3 Fig — Boxplot of the distribution of TIS prediction accuracy values for the genomes in the GOLD database [39], grouped according to sequencing center. (TIF) [file pone.0133691.s003.tif]

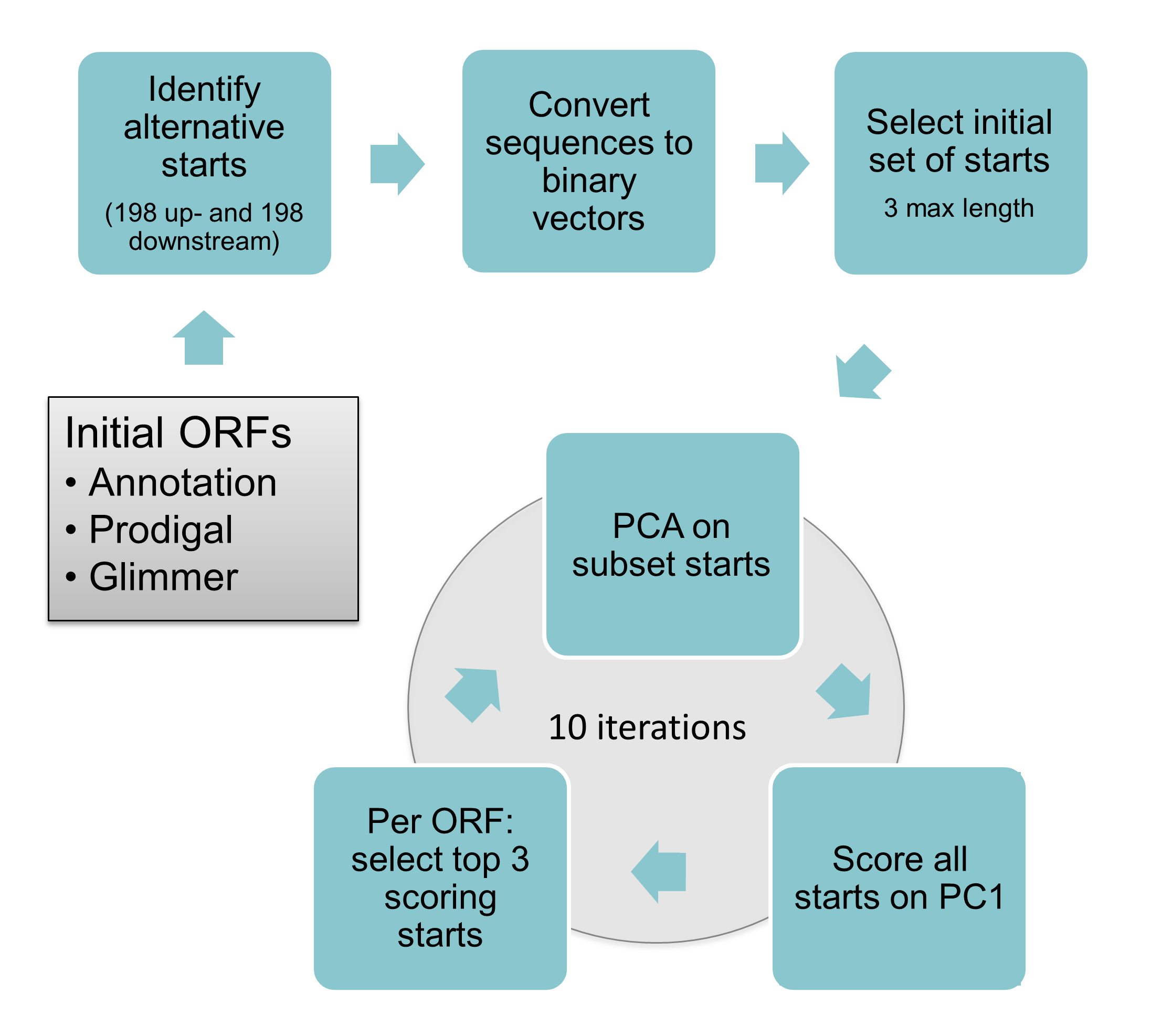

Supplement: S4 Fig — (TIF) [file pone.0133691.s004.tif]

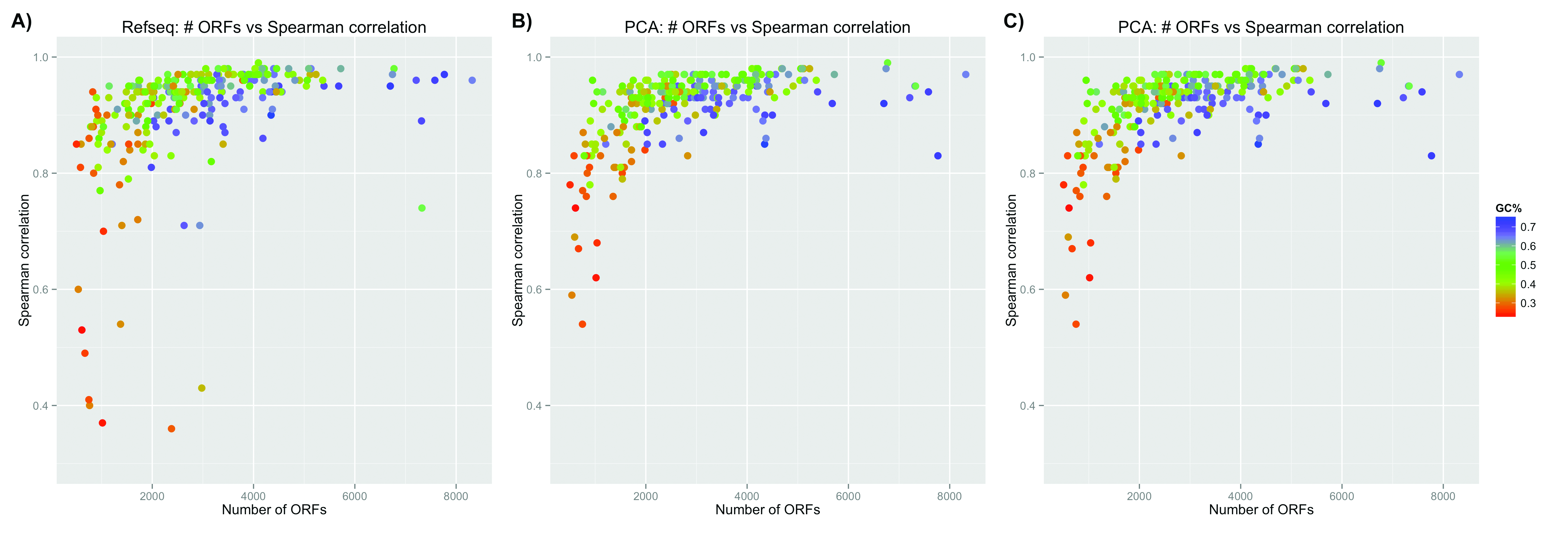

Supplement: S5 Fig — Scatterplot showing the relationship between TIS annotation quality (i.e. the correlation between observed alternative start codon frequencies and expected alternative start codon frequencies) (Y axis) and number of ORFs in a genome (X axis) for 277 bacterial and archaeal genomes ([4]). (TIF) [file pone.0133691.s005.tif]
